# Factors Associated With Discussing High Blood Pressure Readings in Clinical Notes

**DOI:** 10.1093/ajh/hpae153

**Published:** 2024-12-11

**Authors:** Cole G Chapman, Philip M Polgreen, Manish Suneja, Barry L Carter, Linnea A Polgreen

**Affiliations:** Department of Pharmacy Practice and Science, University of Iowa, Iowa City, Iowa, USA; Department of Internal Medicine, University of Iowa, Iowa City, Iowa, USA; Department of Internal Medicine, University of Iowa, Iowa City, Iowa, USA; Department of Pharmacy Practice and Science, University of Iowa, Iowa City, Iowa, USA; Department of Pharmacy Practice and Science, University of Iowa, Iowa City, Iowa, USA

**Keywords:** blood pressure, hypertension, notes, specialty

## Abstract

**BACKGROUND:**

Blood pressure (BP) is routinely measured and recorded at healthcare visits, but high BP (HBP) measurements are not always discussed in clinical notes. Our objective was to identify patient- and visit-level factors associated with discussion of HBP measurements in clinical notes, among patients without prior diagnosis of hypertension.

**METHODS:**

Data from 2016 to 2022 for all patients with any BP record of 140/90 mmHg or greater were obtained from University of Iowa Hospitals and Clinics electronic medical records. Patients with any prior hypertension diagnosis were excluded. We used a multi-level regression model to evaluate differences in the rates of discussing HBP. The model included varying intercepts for visit specialty and non-varying slopes and intercepts for patient- and visit-level features.

**RESULTS:**

The final sample included 278,766 outpatient visits for 27,423 patients, of which 61,739 visits had HBP. Only 31% of visits with HBP had associated clinical notes with a discussion of HBP. Even in primary-care-related clinics, HBP measurements were discussed in only 70% of visits. Factors associated with decreased odds of HBP being discussed in clinical notes included fever (OR: 0.46; 95%CI: 0.24–0.86) or external injury or pain (0.84; 0.79–0.90), and a larger number of comorbidities (6+: 0.27; 0.22–0.32). Discussion of HBP in clinical notes was more likely among visits of patients with prior visits where HBP was discussed in clinical notes (12.36; 11.75–13.01).

**CONCLUSIONS:**

We found that discussion of HBP is relatively uncommon. Increasing discussion of hypertension in clinical notes could decrease hypertension-related diagnostic inertia.

## Graphical Abstract

**Graphical Abstract:**
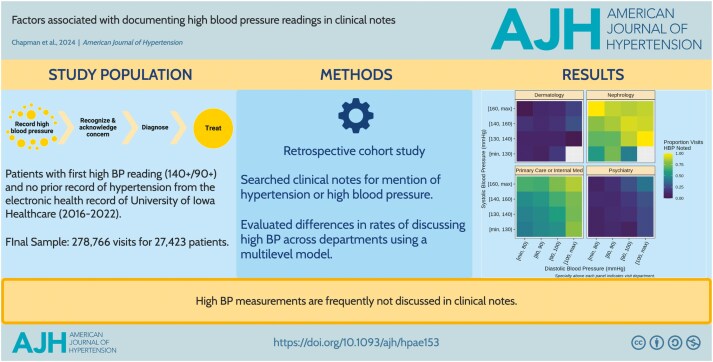
This graphical abstract is also available at Tidbit: https://tidbitapp.io/tidbits/the-proportion-of-physician-notes-addressing-elevated-blood-pressure-readings-varies-dramatically-across-internal-medicine-specialties?utm_campaign=tidbitlinkshare&utm_source=ITP

Hypertension is a leading contributor to morbidity,^1^ second only to smoking as the most significant modifiable cause of death in the United States.^2^ Risks of developing future cardiovascular disease can be substantially reduced with hypertension treatment.^3^ However, hypertension is frequently underdiagnosed and undertreated.^4^ Accordingly, increasing the diagnosis of hypertension is an important public health priority^5^ and new approaches are needed.^6^

Widespread implementation of electronic-health-record systems (EMRs) promised to help improve clinical outcomes for several diseases, including hypertension.^7^ These hopes were bolstered in part by the potential for EMRs to support diagnostic and therapeutic decision-making through increased access to health information across encounters and providers.^8^ However, such broad improvements in outcomes on a national scale have not clearly manifested.^9^ Indeed, progress toward effectively treating hypertension in the United States has actually decreased in recent years.^10^

Blood pressure (BP) measurements are routinely captured and documented in EMRs during healthcare visits.^11^ Unfortunately, high BP (HBP) measurements may not receive adequate attention or documentation in clinical notes.^12^ Clinical notes serve as the primary means of recording and communicating health-related information across various healthcare visits and providers.^13^ Consequently, lack of discussion of HBP measurements in these notes represents a missed opportunity to contribute to the timely identification of hypertension, particularly for patients without regular use of primary healthcare.

The purpose of this analysis was to identify patient- and visit-level factors associated with discussion of HBP in clinical notes. We focus specifically on patient visits with HBP measurements without prior diagnosis of hypertension.

## METHODS

### Data and cohort

Data from The University of Iowa Healthcare EMR, from 01 January 2016 through 31 December 2022, were extracted for all patients with any HBP reading (140+/90+) and no documented diagnosis of primary essential hypertension prior to first HBP during the period. Data elements included patient demographics and records of all visits with the healthcare system, as well as providers, diagnosis codes, and patient vitals including BP. Notes associated with each visit were characterized using a series of regular expressions including the character location for each matching pattern.

Data were aggregated to the visit level for analyses. Visit BP values were measured as the mean of all measurements, after excluding BP records that were incomplete or outside the range 70–280 for systolic and 20–180 for diastolic.

The index HBP for each patient was defined as the first observed HBP during the period from 01 January 2017 to 31 December 2021 to assure a minimum 365 days of data for observation before and after the index HBP event. The observation period for each patient was from 365 days prior to index HBP visit to the earliest occurrence of either a visit with diagnosis of hypertension, death, or the end of the study period (31 December 2022).

Hypertension diagnoses were measured from ICD-10 diagnosis codes, using definitions for both complicated and uncomplicated hypertension from Elixhauser.^14^ Patient-level exclusion criteria (**Table 1**) included age less than 19 at index visit, pre-eclampsia or other pregnancy-related hypertension during the observation period, any inpatient stay ≥30 days, any pre-index visit with HBP, or missing data for variables. Visits with duration greater than 13 hours were excluded from final analyses.

**Table 1.** Consort table

	Observations (*N*)	Patients (*N*)
Patient aged 19+ with any BP 140/90 or greater 2017–2021 and no prior hypertension diagnosis	7,473,273	54,241
Valid BP (systolic 70–280, diastolic 20–180)	6,660,551	54,155
Aggregated to visit-level average BP	890,544	54,155
Has visit BP 140/90 or higher in 2017–2021	635,876	34,613
Visit begin date on or prior to date of any visit with an associated diagnosis of hypertension, or end of follow-up period	374,430	34,613
No hypertension diagnosis during 365 days before index	354,790	32,664
No pregnancy-related hypertension diagnosis during observation period	338,770	31,821
Non-missing patient BMI, visit type, visit admit type, and visit specialty	332,943	30,315
No visit during the observation period with length of stay greater than 30 days	326,667	30,090
No visit with HBP prior to index	303,170	28,880
Non-inpatient visits with duration <24 hours	278,766	27,423
Visit has mean BP of 140/90 or higher	61,739	27,423

### Measures

#### Discussion of BP in clinical notes

The dependent variable of interest was a binary measure of whether HBP or hypertension discussion was present in clinical notes for a patient visit. We used regular-expression-pattern matching to create measures indicating the presence and location of specific terms. Term sets were created for BP (e.g., HTN, blood pressure) and for contextual qualifiers (e.g., high, elevated). Term sets for unrelated hypertension conditions (portal, pulmonary ocular) were used for exclusion. Further details on the algorithm used to identify discussion in notes are given in Supplementary Appendix 1.

#### High blood pressure

We defined HBP as a visit-level-mean systolic BP ≥ 140 mm Hg and mean diastolic BP ≥ 90 mm Hg.

#### Patient and visit characteristics

Visit specialty was determined from the primary department associated with each visit. For visits with unknown department or specialty, the specialty of the primary provider was used. Specialties that represented less than 0.1% of all visits in the sample were combined in an “other specialty” group.

A variable for duration of the visit included categories for visits <3 hours, between 3 and 6 hours, between 6 and 13 hours, and greater than 13 hours. Visits longer than 24 hours were excluded from the main analyses. Binary variables were created to indicate whether the visit was (i) ancillary (e.g., lab only, nurse only), (ii) related to pain or injury (e.g., trauma, poisoning), (iii) related to acute illness (e.g., upper respiratory infection), and/or (iv) included diagnosis of HBP that is not hypertension (ICD-10 diagnosis R03.0).

Patient-level-demographic characteristics included race and gender. Race categories representing less than 2% of the patient sample were labeled, “other or unknown.” Visit-level-patient characteristics were also measured. A longitudinal variable indicating whether the patient had any prior visit with a clinical note discussing HBP was created to capture the potential relevance of information exchange across time. A binary variable indicating whether the patient had a fever at the visit (i.e., body temperature exceeded 101.3F) was created and coded to 0 when no temperature was available. Patient BMI was measured from the most recent height and weight values available for each visit. Patients with no measured height and weight during their observation period were excluded. Patient age was measured for each visit based on birthdate and date of the visit. Diagnosis codes from all visits occurring during the 365 days prior to each visit were used to measure comorbidity burden: a categorical variable indicating a number of distinct Elixhauser comorbid conditions^14^ recorded in the pre-365 period.

### Analyses

Summary statistics for all study variables were computed across patients in the final study cohort at the time of their index HBP visit, across all outpatient visits, and across the subset of HBP visits used for final analyses. Median and interquartile range are reported for all continuous variables, while a count and percent of total are reported for categorical variables. Difference in mean systolic and diastolic BP was tested across visits with and without discussion in clinical notes, and across visits with and without a hypertension diagnosis occurring, using a Welch two-sample t-test. We also tested differences in the proportion of visits with hypertension diagnosis by whether there was discussion in notes using a two-sample test for equality of proportion with continuity correction. Prior to limiting the sample to HBP visits, we calculated variation in rates at which BP was discussed in clinical notes by department specialty, at different levels of BP.

Our primary analysis used a multi-level-regression model to evaluate differences in the rates of discussing HBP in notes across visits. Data used for model fitting included only the HBP visits (140+/90+). The dependent variable was a binary indicator for whether any notes associated with the visit had discussion of HBP or hypertension. Explanatory variables of interest were binary variables indicating any prior visit with notes discussing HBP and whether the visit had a diagnosis for non-hypertension HBP. Additional explanatory variables modeled as fixed effects included visit duration and time, ancillary visit, diagnoses on the visit, comorbidity counts, and patient age, BMI, race, and gender. Model random effects included varying intercepts for visit specialty. Bayesian estimation of the logistic mixed-effects model was accomplished using Stan via the `rstan` package for R.^15^Supplementary Appendix 2 provides further details on model specification. Coefficients were estimated from the median of the posterior distribution and 95% credible intervals were estimated from the 2.5% and 97.5% percentiles, respectively. Model fit and accuracy were evaluated across 1,000 posterior prediction draws. All analyses were carried out in R (version 4.4.1).^16^

Finally, new hypertension guidelines were published in November 2017,^17^ during our study period. Thus, we repeated our analyses using a BP threshold of 130+/80+.

## RESULTS

There were 278,766 visits with BP, for 27,423 distinct patients, that met all criteria for inclusion. Of these, 61,739 visits had HBP (140+/90+) and were used for model estimation. See **Table 1** for exclusion-criteria effects.

Cohort summary statistics are shown in **Table 2**. Race was “White” for 85% of patients in the sample. Most patients did not have any Elixhauser comorbid conditions at the time of their index visit (63%) while approximately 28% had 1–2 comorbid conditions and 9% had 3 or more. Approximately 22% of patients had a diagnosis of pain or injury on their index visit and 9% of patients had a visit diagnosis for an acute illness.

**Table 2.** Cohort summary statistics

Characteristic	Summary statistic^a^ (*N* = 27,423 Patients)
*N* [Visits]	6 (3, 13)
*N* [Visits] with HBP (140+/90+)	2 (1, 3)
Systolic BP (index visit)	151 (145, 160)
Diastolic BP (index visit)	94.1 (92.0, 99.0)
High BP in clinical note (index visit)	7,831 (29%)
Race	
White	23,265 (85%)
Black or African American	1,989 (7.3%)
Hispanic	835 (3.0%)
Other or unknown	1,334 (4.9%)
Gender	
Female	12,022 (44%)
Male	15,401 (56%)
Age at index visit (years)	
<30	3,426 (12%)
(30, 45)	7,572 (28%)
(45, 60)	8,928 (33%)
60+	7,497 (27%)
Body mass index (index visit)	
<25	5,044 (18%)
(25, 35)	14,821 (54%)
35+	7,558 (28%)
Comorbidity count (index visit)	
0	17,181 (63%)
1	5,173 (19%)
2	2,479 (9.0%)
3	1,262 (4.6%)
4–5	983 (3.6%)
6+	345 (1.3%)
Visit diagnosis (index)	
Hypertension	2,263 (8.3%)
Non-hypertension HBP	1,205 (4.4%)
Pain or external cause/injury	5,911 (22%)
Acute illness	2,445 (8.9%)
Pre-index	
Diagnosis, non-hypertension HBP	135 (0.5%)
High BP in clinical note	4,427 (16%)
Fever (101.3 F)	27 (<0.1%)
Post-index	
High BP in clinical note	19,592 (71%)
Diagnosis, hypertension	11,159 (41%)

^a^Median (IQR) for continuous measures; *n* (%) for categorical measures.

Patients in the sample had a median of 6 (IQR: 3–13) total visits and 2 (IQR: 1–3) visits with HBP. The distribution of number of visits with HBP was strongly skewed: 72% of patients had 1–2 HBP visits during their observation period while 23% had 3–5, 5% had 6–15, and 0.2% had 16 or more.

HBP was discussed in the index visit clinical notes for 29% of patients and 16% of patients had some discussion in a note prior to the index visit. Mean BP level on patient’s index visit was greater for patients with discussion in their clinical notes (155/97) compared to those without discussion (154/96), a difference that was statistically (*P* < 0.001) but not clinically significant. A new diagnosis of hypertension occurred on the index visit with first HBP for 8.3% of the cohort and was more common among patients with discussion in clinical notes (19%) than those without (4%). Mean BP on index visits with a hypertension diagnosis was 161/99, compared to 154/96 among those without a diagnosis. By the end of the observation period, 71% of all patients had at least 1 visit with discussion of HBP in clinical notes, and 41% of patients received a hypertension diagnosis.

### High blood pressure visits across departments

The percent of visits with HBP was greatest for the Emergency Department (44.8%), followed by Ophthalmology (44.7%), Audiology (41.2%), Transplant (36.0%), Dentistry (35.3%), Orthopedics (35.2%), and Urgent Care (34.9%). The percentage of HBP visits having HBP mentioned in clinical notes was greatest for Nephrology (90.9%), followed by Primary Care or Internal Medicine (69.7%) and Urgent Care (52.4%). Conversely, mention of HBP in clinical notes was rarest among visits with Infusion Therapy (0.5%), Radiology (0.5%), and Audiology (1.7%).

### Discussion of high blood pressure as blood pressure increases

**Figure 1.**
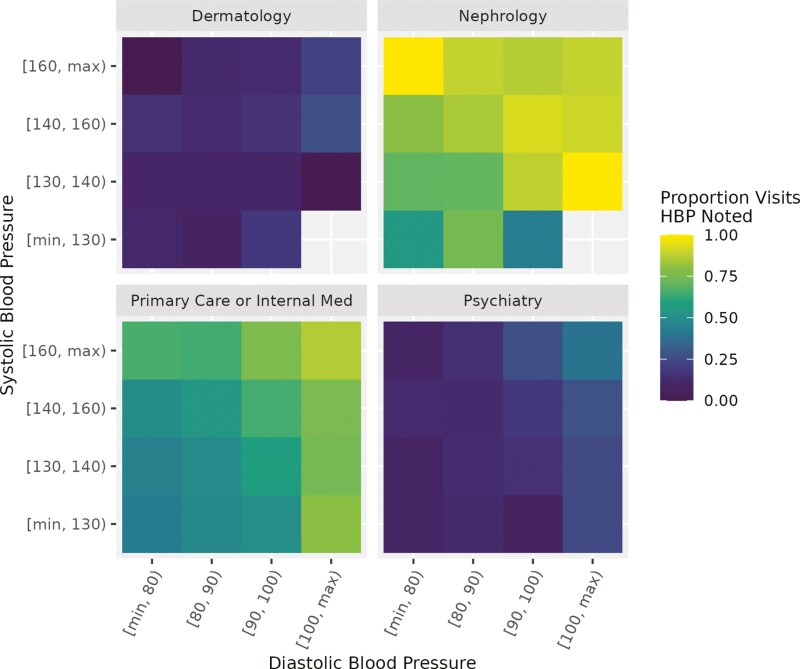
Specialty discussion of high blood pressure (BP) by BP level.

**Figure 1** shows selected specialty results by BP level. The color of each tile represents the proportion of visits at a particular BP level that had discussion of HBP or hypertension in clinical notes; from dark purple (low) to bright yellow (high). Systolic BP is on the *y*-axis and diastolic BP is on the *x*-axis.

Among visits with “Primary Care or Internal Medicine,” increased discussion of HBP occurred with greater BP values. Among visits with Nephrology, discussion of HBP in notes was generally greater across BP-level combinations, but the proportion was greatest among visits with BP values in ranges of 130–139/100+ or 160+/<80. Conversely, an overall lower proportion of visits with Psychiatry had any discussion of HBP in clinical notes but similar trend of increasing discussion with rising BP. For many specialty groups, particularly those with low overall rates of discussion of HBP in clinical notes, there was no clear or consistent association between higher BP and percent of visits with discussion in clinical notes. For example, visits with Dermatology do not show a consistent trend of increasing discussion with any level of rising systolic or diastolic BP.

#### Model estimates

**Figure 2.**
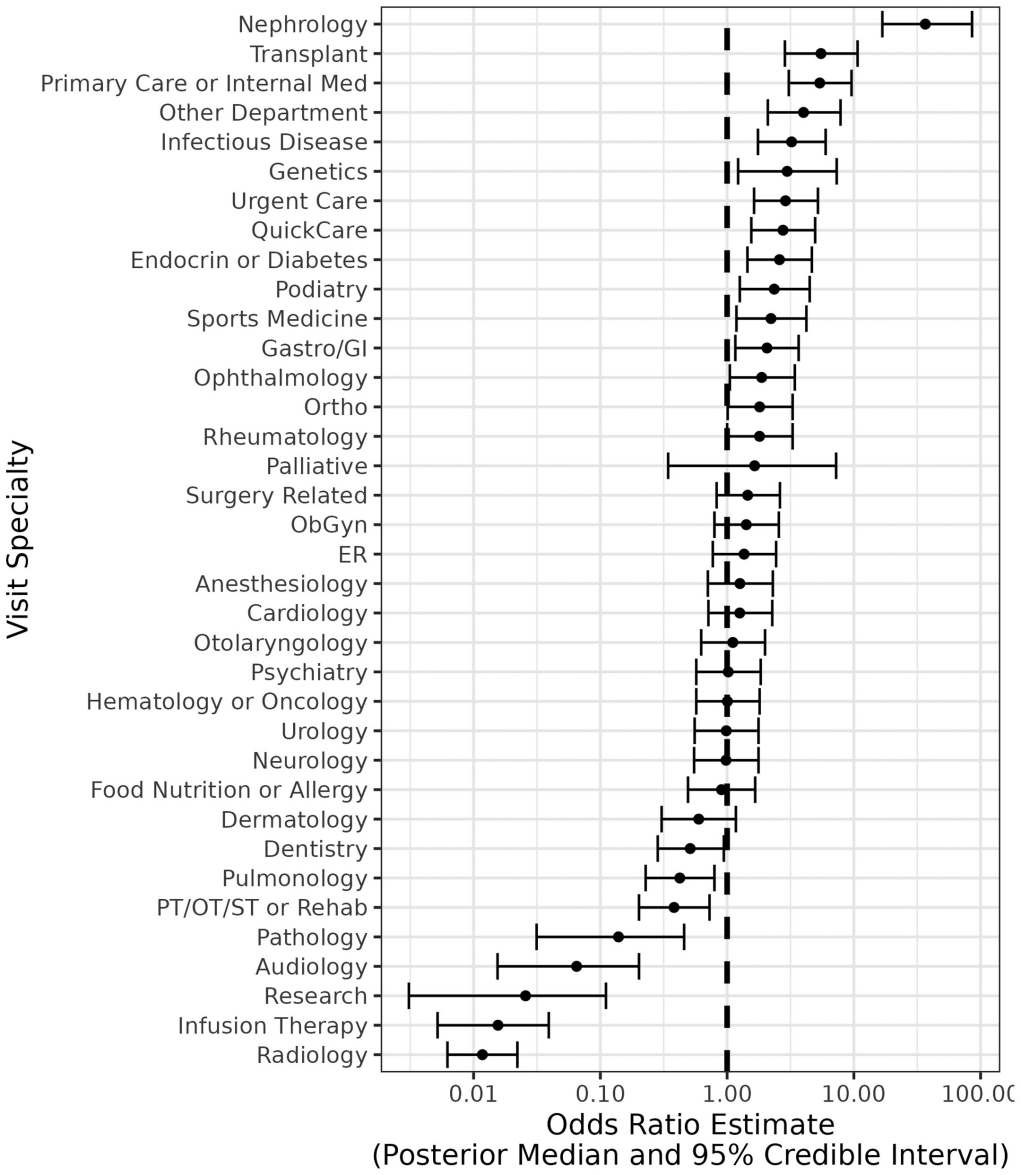
Odds of discussion of high BP in the clinical note by specialty.

**Table 3** shows estimated parameters from our multi-level model predicting whether the clinical notes for a visit included discussion of HBP or hypertension. Odds ratios and 95% credible interval estimates for visit specialty varying intercept terms are included in **Figure 2**. The estimated model Bayes *R*^2^ value was 0.41.

**Table 3.** Predictors of high BP discussion

	Dependent variable: high BP discussed in clinical note	
Predictors	Odds ratios^a^	95% CI^b^
Intercept	0.14	0.08–0.25
Prior clinical note of HBP	12.36	11.75–13.01
Visit diagnosis, non-hypertension HBP	8.87	7.83–10.08
Visit duration (hours)		
<3	Reference	
(3, 6)	0.97	0.91–1.03
(6, 13)	0.91	0.83–1.00
(13, 24)	1.29	1.13–1.48
Ancillary visit	0.09	0.07–0.12
Visit time 11:00 am–12:00 pm	0.82	0.75–0.90
Visit diagnosis, pain or external cause	0.84	0.79–0.90
Visit diagnosis, acute illness	0.91	0.84–0.99
Fever	0.46	0.24–0.86
Comorbidity count		
0	Reference	
1	0.59	0.56–0.63
2	0.46	0.43–0.50
3	0.39	0.36–0.44
4–5	0.37	0.33–0.41
6+	0.27	0.22–0.32
Age at visit		
(18, 30)	Reference	
(30, 45)	1.19	1.11–1.29
(45, 60)	1.39	1.28–1.50
60+	1.46	1.34–1.58
BMI (centered, scaled by 1 SD)	1.08	1.06–1.11
Race		
White	Reference	
Black/African American	1.16	1.07–1.26
Hispanic	0.93	0.81–1.06
Other or unknown	1.11	1.00–1.23
Male gender	0.88	0.84–0.92
Model fit (posterior prediction)^c^		
Sensitivity	0.59	
Specificity	0.82	
Positive predictive value	0.77	
Negative predictive value	0.67	
Accuracy	0.71	
Bayes *R*^2^	0.41	
Bayes *R*^2^, fixed effects only	0.27	
Observations	61,739	

^a^Median value across posterior sample size of 36,000.

^b^95% credible interval from 2.5th and 97.5th percentiles of the posterior distribution.

^c^Median value across 1,000 posterior prediction draws for each observation in the data. Bayes *R*^2^ calculated as the outcome variance divided by summed outcome and residual variance.

The odds that HBP was discussed in clinical notes were 12.4 times greater (95% CI: 11.8–13.0) among visits with a patient who had an earlier visit with notes discussing HBP, despite no actual diagnosis of hypertension having occurred for these patients. Similarly, odds were 8.9 times greater (7.8–10.1) among visits where non-hypertension HBP was a diagnosis code associated with the visit.

Discussion of HBP was much less likely (OR 0.09, 95% CI 0.07–0.12) for ancillary visits. Discussion was also less likely for visits occurring just prior to the typical lunch hour between 11:00 am and 12:00 pm and for visits with problems of acute illness, fever, external injury, or pain. Presence of comorbidities was associated with less discussion of HBP; a single comorbidity was associated with 0.59 times lower odds (0.56–0.63) relative to no comorbidity, and 6+ comorbidities were associated with 0.27 times lower odds (0.22–0.32).

**Figure 2** shows the estimated varying intercepts for visit specialty, with bars representing 95% credible intervals. The odds of notes including discussion of HBP or hypertension was 36.6 times (16.8–85.6) greater among HBP visits with Nephrology and 5.4 times greater (3.1–9.6) among HBP visits with Primary Care or Internal Medicine. Conversely, odds were approximately 20 times lower (OR 0.06; 95%CI 0.02–0.20) among visits with Audiology.

Results using a 130+/80+ threshold were similar to the results presented here. See Supplementary Appendix 3.

## DISCUSSION

We found that BP and hypertension were not routinely discussed in the clinical notes for patient visits with HBP (140/90 and higher). Across all visits with HBP recorded, only 31% had any associated clinical note with a corresponding discussion of BP or hypertension. Discussions of HBP measurements in notes varied widely based on medical specialty, but even in primary-care-related clinics, HBP measurements were mentioned in only 70% of visits. High BP was less likely to be discussed in notes of visits with patients presenting with acute problems (e.g., pain, injury, infection). In contrast, visits for patients with prior reference to BP or hypertension outside the vital signs portion of the note, or a prior diagnosis of non-hypertension HBP were approximately 10 times more likely to have a reference of BP or hypertension in their clinical note.

Currently, in the absence of end-organ disease, multiple BP readings are needed for a diagnosis.^18^ Contributing to the diagnostic inertia commonly associated with hypertension is the practice of attributing high measurements to “white coat” hypertension.^19^ Also, clinic measurement of BP may be suboptimal, and providers might discount high values. To overcome these barriers to hypertension diagnosis, interventions have focused on measuring BP outside of traditional healthcare settings. Examples include health fairs,^20^ churches,^21^ and barber shops.^22^ Other interventions have focused on increasing home readings.^23,24^ However, these interventions require transferring measurements back to healthcare providers and subsequent follow-up with patients. In contrast, the EMR provides a convenient platform for recording and viewing BP values. Yet, despite the ubiquity of EMRs and the near-universal recording of BP measurements at clinic visits, hypertension diagnosis and treatment remain suboptimal.^10^ EMR-based automatic alerts to address hypertension could be implemented, but they have had limited success in previous studies.^25–27^

We hypothesize that a reference to BP outside of the vital signs section of the note is a marker for recognition. Indeed, discussing smoking in the clinical record is associated with future smoking-related conversations between physicians and patients.^28^ Also, including obesity in a clinical note is associated with initiating obesity-related therapy.^29^ While we cannot claim that discussion of HBP will directly lead to the diagnosis of hypertension and better BP control, we found that patients with discussion of HBP in clinical notes were more likely to have additional BP discussions included in the notes of subsequent visits. Furthermore, patients previously diagnosed with “elevated blood pressure, not hypertension” were substantially more likely to have BP referenced in future notes.

Our results support two reasons for failure to discuss HBP measurements. First, healthcare providers are busy and confronted with competing clinical problems.^30^ Indeed, notes were less likely to reference BP measurements if patients presented with an acute problem (e.g., infection or injury). Additionally, patients with multiple comorbidities were less likely to have clinical notes referencing BP. Having multiple medical problems likely translates into less time for focusing on HBP measurements. Furthermore, notes were less likely to reference BP when patients presented just before lunchtime. These patients may have less time dedicated to their visit to focus on BP because of prior patient visits running overtime compared to patients with clinic visits earlier in the day.

A second factor contributing to the lack of discussion of HBP readings in clinical notes may stem from healthcare providers perceiving the diagnosis of hypertension as beyond their specific responsibilities. For example, clinics with a primary focus on hypertension management, such as nephrology and primary care, exhibit significantly higher rates of including BP discussions in notes, in stark contrast to medical specialties less directly associated with hypertension, like psychiatry and dermatology. Interestingly, even though cardiologists and neurologists are expected to possess advanced knowledge about hypertension, our findings indicate that visits associated with these specialties do not significantly differ from those in infectious diseases and gastroenterology concerning the discussion of HBP readings. This suggests that factors beyond potential knowledge disparities may contribute to the variability in discussing abnormal BP readings across different medical specialties.

Our study has several limitations. First, this study was conducted at one institution, and though we believe that the results would be similar in other institutions, the results might not be generalizable. However, the methods we propose here can be implemented at any institution with an EMR. Second, we used “hypertension” and “BP” as a marker for discussions of HBP. These terms may refer to other diseases, but we controlled for cases of portal, pulmonary, or ocular hypertension. Nevertheless, some notes might have been misclassified. For example, some discussions could have referred to a family history of hypertension. However, such misclassifications would bias our results toward the null, providing a more optimistic measure of BP or hypertension discussions in clinical notes. Third, BP may have been discussed but not recorded.^31^

Despite our limitations, we found that HBP measurements are often recorded but seldom discussed in clinical notes, especially outside primary-care settings. Our results identify a new opportunity to overcome hypertension-related diagnostic inertia: improving discussions in clinical notes. While efforts to increase the measurement of BP outside clinic settings should be encouraged, our results highlight the importance of recognizing HBP values currently recorded in the EMR across different clinical settings.

## Supplementary material

Supplementary materials are available at *American Journal of Hypertension* (http://ajh.oxfordjournals.org).

hpae153_suppl_Supplementary_Appendix

## Data Availability

For patient privacy reasons, research data cannot be shared.
