# Supplementary material for: Factors Associated With Discussing High Blood Pressure Readings in Clinical Notes
Source: Am J Hypertens. 2024 Dec 11;38(4):225–32. doi: 10.1093/ajh/hpae153 (PMC11911316; doi:10.1093/ajh/hpae153)

Appendix 1: Creating the notes variable.

The dependent variable of interest was a measure of whether providers discussed high BP or concerns of hypertension in the clinical notes associated with a patient visit. This binary variable was measured at a visit-level and equaled 1 if any note associated with the visit discussed high BP or hypertension, 0 otherwise. To create this measure, we first used regular-expression-pattern matching to create note-level measures indicating presence and location of terms within a specified set. Term-sets were built to identify each of the following elements in clinical notes:

1. section heading (e.g. “Comments”, “Impressions”)
2. BP (e.g. “blood pressure”, “BP”)
3. qualifications of BP severity or magnitude (e.g. “high”, “elevated”)
4. hypertension (e.g. “htn”, “hypertension”)
5. other hypertension not related to study objectives (e.g. “portal”, “pulmonary”, “ocular”)

For each term-set, matches returned an integer value indicating the character position of the match within the note. A rule-based algorithm, illustrated in Appendix Figure 1, was then applied to the term-set results for each note to create a measure of whether high BP was discussed.

Notes with a match to the ‘BP’ and/or ‘hypertension’ term-sets were considered as candidates indicating discussion of high BP. A rule-based algorithm was then applied to these candidate notes to determine relevance and address possible misclassification. For matches in the ‘BP’ term-set, a qualifier term (e.g. “high”) was required to be located within 50 characters. Matches in the ‘hypertension’ term-set were disqualified if any match from the ’other hypertension’ term-set (e.g. “portal”) was located within 100 characters, or if there was any clinical diagnosis code for an ‘other-hypertension’ condition associated with the visit. Finally, for note types and sources containing multiple sections, candidate matches were required to be located after found matches for section headings that were appropriate to the target context (“Impression”, “Comments”).

Appendix Figure 1: Algorithm for determining whether discussion of high BP was contained in the clinical note.

**
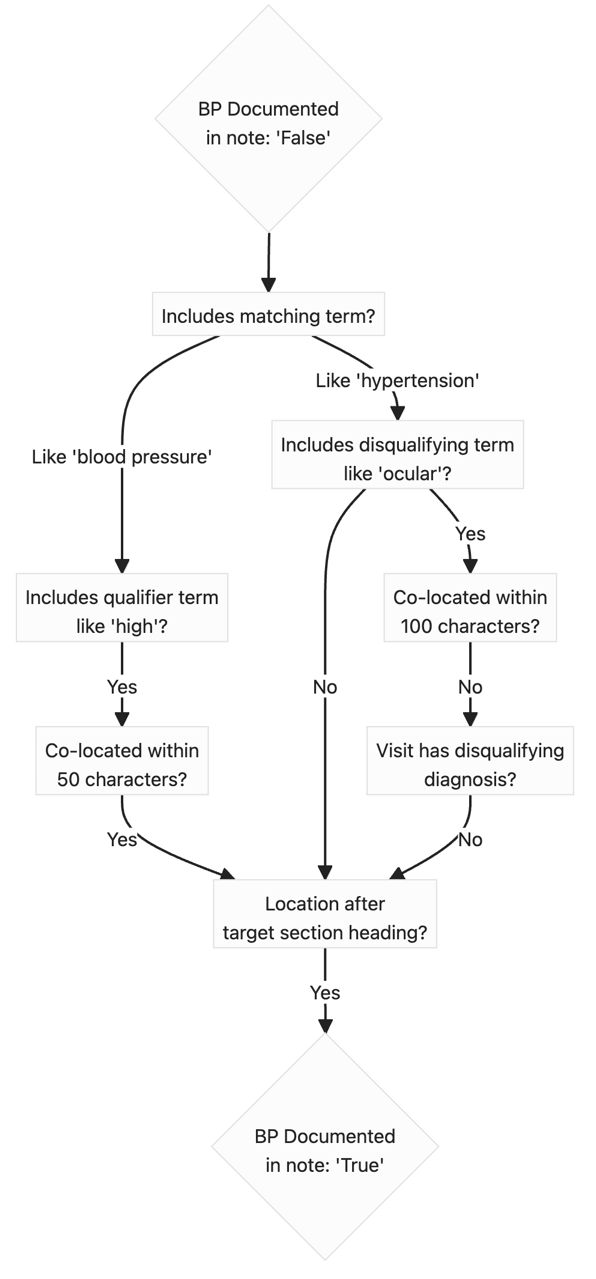
**

Appendix 2: Primary model estimation

Our primary analysis used a multi-level logistic regression model to evaluate differences in the rates of discussing high blood pressure (HBP) in clinical notes across visits where HBP was present, where HBP was defined as BP of 140+/90+. The model included varying intercepts for visit specialty and non-varying slopes and intercepts for other patient and visit features. The following model was specified and estimated following a fully Bayesian framework:

$${Note}_{ij}=\beta_{0}+\beta_{X}\cdot[X_{ij}^{PriorNote}+X_{ij}^{DxHBP}+X_{ij}^{VisitDuration} +X_{ij}^{AncillaryVisit}+X_{ij}^{VisitTime}+X_{ij}^{DxPainInjury}+X_{ij}^{DxAcuteIllness}+X_{ij}^{Fever}+X_{ij}^{NComorb}+X_{ij}^{Age}+X_{ij}^{BMI}+X_{i}^{Race}+X_{i}^{Gender}]+\alpha_{0,S}\cdot S_{ij}$$

The Bernoulli response variable, ${Note}_{ij}$, indicated whether high BP or hypertension was found in the clinical notes associated with visit $j$, for patient $i$. The model’s random effects included varying intercept terms, $\alpha_{0,S}$, estimated for visit specialty, $S$. The model’s fixed effect estimates included the model intercept term, $\beta_{0}$, and a vector of non-varying slope coefficients ($\beta_{X}$) with terms corresponding to sets of variables for patient- and visit-level characteristics (represented by $X_{i}^{*}$ and $X_{ij}^{*}$, respectively).

All analyses were carried out using R statistical software.^1^ Models were estimated using Markov Chain Monte Carlo (MCMC) with Stan (2.26.1),^2^ via the R package ‘rstan’.^3^ Sampling for estimation included 18-chains of 4,000 samples each (2,000 warm-up, 2,000 sampling). Final estimates were therefore based on a total posterior sample size of 36,000.

1. *R: A language and environment for statistical computing* [computer program]. Version 4.3.1. Vienna, Austria: R Foundation for Statistical Computing; 2023.

2. *Stan Modeling Language Users Guide and Reference Manual* [computer program]. Version 2.26.12024.

3. *RStan: the R interface to Stan* [computer program]. Version R package version 2.32.32023.

Appendix 3: Model results with 130/80 mm Hg threshold

Appendix Table 1 shows estimated parameters from our multi-level model predicting whether the clinical notes for a visit included discussion of HBP or hypertension using a threshold of 130/80 mm Hg. The estimated model Bayes R^2^ value was 0.41.

*Appendix Table 1 Predictors of High BP Discussion*

|  | **Dependent Variable:**  **High BP Discussed in Clinical Note** | |
| --- | --- | --- |
| ***Predictors*** | ***Odds Ratios^1^*** | ***95% CI*^2^** |
| (Intercept) | 0.1 | 0.06 - 0.17 |
| Prior Clinical Note of HBP | 13.83 | 13.38 - 14.31 |
| Visit Diagnosis, Non-Hypertension HBP | 10.64 | 9.64 - 11.76 |
| Visit Duration (Hours) |  |  |
| <3 | *Reference* |  |
| [3, 6) | 0.99 | 0.95 - 1.04 |
| [6, 13) | 0.9 | 0.85 - 0.96 |
| [13, 24) | 1.57 | 1.42 - 1.73 |
| Ancillary Visit | 0.08 | 0.06 - 0.1 |
| Visit Time 11:00a – 12:00p | 0.84 | 0.79 - 0.89 |
| Visit Diagnosis, Pain or External Cause | 0.88 | 0.84 - 0.92 |
| Visit Diagnosis, Acute Illness | 0.92 | 0.88 - 0.97 |
| Fever | 0.77 | 0.51 - 1.16 |
| Comorbidity Count |  |  |
| 0 | *Reference* |  |
| 1 | 0.62 | 0.59 - 0.64 |
| 2 | 0.48 | 0.46 - 0.51 |
| 3 | 0.4 | 0.38 - 0.43 |
| 4 - 5 | 0.38 | 0.36 - 0.41 |
| 6+ | 0.3 | 0.27 - 0.33 |
| Age at Visit |  |  |
| [18, 30) | *Reference* |  |
| [30, 45) | 1.16 | 1.11 - 1.22 |
| [45, 60) | 1.32 | 1.26 - 1.39 |
| 60+ | 1.34 | 1.27 - 1.41 |
| BMI (centered, scaled by 1 SD) | 1.08 | 1.06 - 1.09 |
| Race |  |  |
| White | *Reference* |  |
| Black/African American | 1.08 | 1.02 - 1.15 |
| Hispanic | 0.94 | 0.86 - 1.03 |
| Other or Unknown | 1.04 | 0.97 - 1.11 |
| Male Gender | 0.89 | 0.86 - 0.92 |
| ***Model Fit (Posterior Prediction)^3^*** | | |
| Sensitivity | 0.57 | |
| Specificity | 0.84 | |
| Positive Predictive Value | 0.78 | |
| Negative Predictive Value | 0.66 | |
| Accuracy | 0.71 | |
| Bayes *R^2^* | 0.41 | |
| Bayes *R^2^*, fixed effects only | 0.26 | |
| ***Observations*** | 145,305 | |
| *^1^* Represents the median value of the distribution of posterior draws.  *^2^* 95% credible interval: represents the 2.5^th^ and 97.5^th^ percentiles of the distribution of posterior distribution. *^3^* Median value across 1,000 posterior prediction draws for each observation in the data. Bayes *R^2^* calculated as the outcome variance divided by summed outcome and residual variance. | | |

Appendix Figure 2: Varying intercepts for visit specialty in our multi-level model, with bars representing 95% certainty intervals.


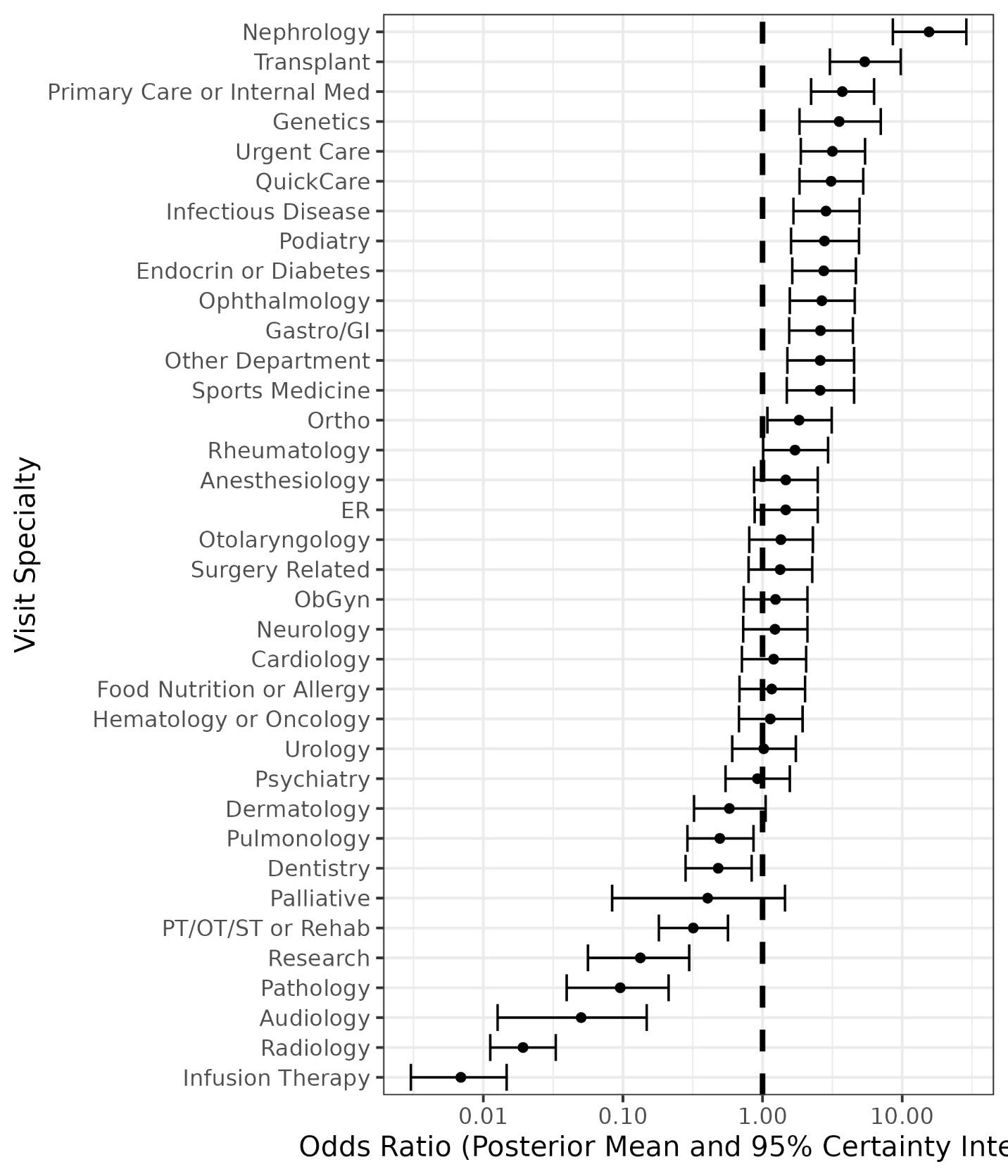

Supplement: hpae153_suppl_Supplementary_Appendix [file hpae153_suppl_supplementary_appendix.docx]
